# Advanced Magnetic Resonance Imaging in the Evaluation of Treated Glioblastoma: A Pictorial Essay

**DOI:** 10.3390/cancers15153790

**Published:** 2023-07-26

**Authors:** Matia Martucci, Rosellina Russo, Carolina Giordano, Chiara Schiarelli, Gabriella D’Apolito, Laura Tuzza, Francesca Lisi, Giuseppe Ferrara, Francesco Schimperna, Stefania Vassalli, Rosalinda Calandrelli, Simona Gaudino

**Affiliations:** 1 Dipartimento di Diagnostica per Immagini, Radioterapia Oncologica ed Ematologia, Fondazione Policlinico “A. Gemelli” IRCCS, 00168 Rome, Italy; rosellina.russo@policlinicogemelli.it (R.R.); carolina.giordano@guest.policlinicogemelli.it (C.G.); chiara.schiarelli@policlinicogemelli.it (C.S.); gabriella.dapolito@policlinicogemelli.it (G.D.); rosalinda.calandrelli@policlinicogemelli.it (R.C.); simona.gaudino@policlinicogemelli.it (S.G.); 2 Istituto di Radiologia, Università Cattolica del Sacro Cuore, 00168 Rome, Italy; laura.tuzza01@icatt.it (L.T.); francesca.lisi14@icatt.it (F.L.); giuseppe.ferrara07@icatt.it (G.F.); francesco.schimperna01@icatt.it (F.S.); vassallistefan@libero.it (S.V.)

**Keywords:** brain tumor imaging, gliomas, treatment-related changes, advanced MR imaging, perfusion MRI, MR spectroscopy, AI

## Abstract

**Simple Summary:**

Glioblastoma is the most common malignant primary tumor of the central nervous system, with a poor prognosis despite many available treatments, including surgery, radiotherapy, and chemotherapy. The evaluation of treatment response is essential to optimize patient outcomes. While structural MRI remains the cornerstone of imaging evaluation, advanced MRI modalities have increasingly become crucial in characterizing treatment effects more comprehensively. The purpose of this pictorial essay is to provide an overview on the role of advanced MRI modalities at the different clinical-therapeutic timepoints, thus helping radiologists and clinicians to be more confident in their applicability in clinical practice and at the proper timepoint.

**Abstract:**

MRI plays a key role in the evaluation of post-treatment changes, both in the immediate post-operative period and during follow-up. There are many different treatment’s lines and many different neuroradiological findings according to the treatment chosen and the clinical timepoint at which MRI is performed. Structural MRI is often insufficient to correctly interpret and define treatment-related changes. For that, advanced MRI modalities, including perfusion and permeability imaging, diffusion tensor imaging, and magnetic resonance spectroscopy, are increasingly utilized in clinical practice to characterize treatment effects more comprehensively. This article aims to provide an overview of the role of advanced MRI modalities in the evaluation of treated glioblastomas. For a didactic purpose, we choose to divide the treatment history in three main timepoints: post-surgery, during Stupp (first-line treatment) and at recurrence (second-line treatment). For each, a brief introduction, a temporal subdivision (when useful) or a specific drug-related paragraph were provided. Finally, the current trends and application of radiomics and artificial intelligence (AI) in the evaluation of treated GB have been outlined.

## 1. Introduction

Glioblastoma (GB) is the most common primary malignant tumor of the central nervous system (CNS), known for its aggressive nature and limited treatment options. The National Comprehensive Cancer Network (NCCN) Guidelines provide evidence-based recommendations for its management.

The standard treatment approach for newly diagnosed GB, as outlined in the NCCN Guidelines, involves a combination of surgical resection, radiotherapy (RT), and chemotherapy with temozolomide (TMZ), commonly referred to as the Stupp protocol. Surgical resection aims to remove as much of the tumor as feasible without causing significant neurological deficits. Following surgery, RT is administered concurrently with adjuvant TMZ, which is an oral chemotherapy agent.

In cases of tumor recurrence, the treatment strategy depends on the location and extent of recurrence. If the recurrence is localized, surgical resection may be considered as an option before initiating systemic therapy.

For diffuse recurrence or cases where surgery is not feasible, systemic therapy becomes the primary treatment choice. The NCCN Guidelines suggest several preferred regimens for recurrent GB, including bevacizumab (an antiangiogenic agent), TMZ, lomustine or carmustine (chemotherapeutic agents), the PCV regimen (a combination of procarbazine, lomustine, and vincristine), and regorafenib (a targeted therapy). The selection of the most appropriate regimen depends on various factors, including the patient’s medical history, previous treatments, and performance status (Table 1) [1].

**Figure 1:**
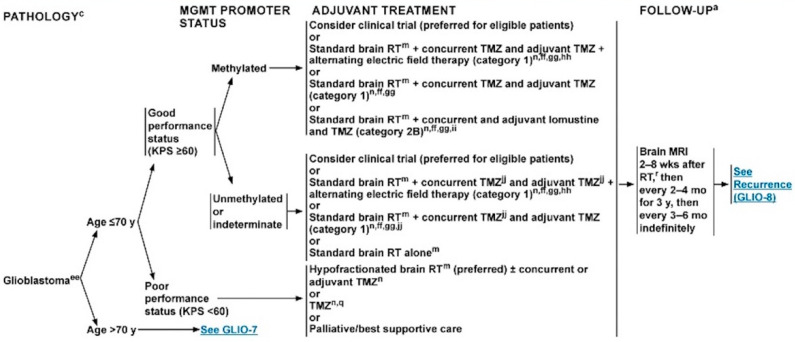
Schematic representation of GB’s management [1].

Magnetic resonance imaging (MRI) plays a key role in the whole clinical history of patients with GB, from the diagnosis to post-surgical evaluation and the monitoring of treatment effects. The NCCN recommends MRI immediately after surgery (up to 48–72 h), 2 to 8 weeks after RT, then every 2 to 4 months for 3 years, then every 3 to 6 months indefinitely (Figure 1) [1]. In case of a second surgery, additional post-operative brain MRI is needed. 

It is well known that the neuroradiological scenario varies depending on the clinical and treatment timepoint and that structural MR imaging alone is often insufficient and unreliable to interpret and define treatment-related changes, such as the pseudophenomena (pseudoprogression and pseudoresponse) or specific drug-related MRI patterns. For that, advanced MRI modalities are increasingly utilized in clinical practice to characterize treatment effects more comprehensively. These include dynamic susceptibility contrast (DSC)- and dynamic contrast enhancement (DCE)-perfusion-weighted imaging; higher order diffusion techniques, such as diffusion tensor imaging (DTI); and MR spectroscopy (MRS).

This pictorial essay focuses on the role of advanced MRI modalities in differentiating various neuroradiological scenarios encountered during the follow-up of treated GBs. Unlike other works, this essay takes a longitudinal approach, reflecting real-life clinical practice. In particular, we identified three main timepoints in the treatment history: post-surgery, during Stupp (first-line treatment), and at recurrence (second-line treatment). For each, a brief introduction, a temporal subdivision (when useful), or a specific drug-related paragraph has been provided. By covering these timepoints, the essay provides insights into the evolving radiological features and challenges encountered during the course of GB treatment. It offers radiologists and clinicians a practical framework to understand the expected changes and interpret the imaging findings in a more informed manner.

Finally, the current trends and application of radiomics and artificial intelligence (AI) in the evaluation of treated GB have been outlined.

## 2. Early Post-Operative Imaging in Glioblastoma 

Surgery undoubtedly represents the main treatment option for patients with newly diagnosed GB, sometimes playing a crucial role even at recurrence. Surgical options at diagnosis can range from a minimally invasive biopsy to a craniotomy with the therapeutic goal of removing as much tumor tissue as safely feasible using microsurgical techniques, without compromising neurological function. The main guiding principles of brain tumor surgery are gross total resection (GTR) when appropriate, minimal surgical morbidity, and accurate diagnosis [1,2].

Several tools, including surgical navigation systems housing functional MRI or diffusion tensor imaging (DTI) datasets and intraoperative MRI, ultrasonography, functional monitoring, and fluorescence-based visualization of tumor tissue with 5-aminolevulinic acid, help in reducing post-operative residual tumor volumes while keeping the risk of new neurological deficits low [2,3]. 

The prognostic impact of the extent of tumor resection (EOR) is actually well established. In fact, a radical surgical approach (GTR) significantly increases survival length and quality when compared with a less radical approach (subtotal resection, STR) [4,5]. Sanai et al. demonstrated that an EOR ≥78% impacts patient outcome, and that this trend continues even at the highest levels of resection [6]. This is also true for recurrent GB, suggesting that patients with initial STR may benefit from surgery with a GTR at recurrence [7]. 

Thus, it is extremely important to determine the EOR as precisely as possible when assessing the results of surgery. Although the EOR was previously estimated by the neurosurgeons [8], it is now well recognized that the radiological detection and quantification of residual tumor is far more sensitive than intraoperative estimation [9]. MRI represents the leading imaging modality, and it is vastly superior to computed tomography (CT) in detecting residual tumor after resection [10]; CT remains an alternative in patients who cannot have an MRI (claustrophobia or unsafe implantable devices).

Time window for post-operative MR imaging has been a critical concern in recent years. 

Different authors have demonstrated that an MRI obtained within the first 3 days after surgery minimized the confounding effects related to post-surgical modifications, in particular nontumoral marginal enhancement (which may mimic residual enhancing tumor), methemoglobin in the surgical bed, and eventually, tumor regrowth [8,10]. In this time window, benign enhancement related to surgical trauma is unusual, even if up to 20% of patients can show an early dural and leptomeningeal enhancement (generally near the craniotomy site or at intergyral and interlobar interfaces). 

**Figure 2:**
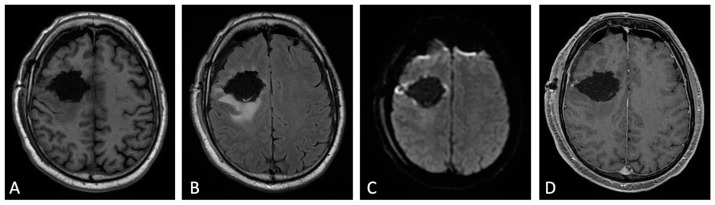
Early post-operative MRI. Axial pre-contrast T1w (**A**), FLAIR (**B**), DWI (**C**), and post-contrast T1w (**D**). Axial FLAIR (**B**) shows mild brain edema around the surgical site. No methemoglobin is seen on pre-contrast T1w (**A**). In DWI (**C**) a thin rim of hyperintensity (probably due to T2w- shine through effect) around the tumor cavity was attributed to post-operative change. The resection margins are free of enhancement except for a small enhancing area on the lateral aspect of surgical cavity compatible with dilated vein (**D**). Dural linear contrast enhancement could be seen in immediate post-operative scan (**D**).

Indeed, a post-surgery baseline MRI scan should ideally be obtained within 24 to 48 h and no later than 72 h [11,12]. The rationale given for performing early post-operative MRI encompasses the assessment of the residual tumor (EOR), but also the detection of surgical complications as early as possible, as well as the availability of a baseline MRI study to plan radiotherapy and to assess treatment response (Figure 2) [8,9,13].

A standardized MR protocol comprehensive of volumetric three-dimensional isotropic pre-contrast and post-contrast T1-weighted images, usually allows for the differentiation of the tumoral enhancement from enhancement due to the expected post-operative changes [14].

If pre-contrast and post-contrast images are carefully compared and anatomic conditions are considered, it is possible to differentiate T1 shortening due to residual enhancing tumor and early methemoglobin during the first 3 days after surgery, and to overcome several other diagnostic pitfalls affecting the evaluation of residual tumor, such as enhancement of the ependymal layer, the choroid plexus, or dilated veins at the operative site.

Uncertain findings exist, such as some kind of ultra-early contrast leakage after traumatic brain laceration. As assumed by Elster et al. [15], a variable, although not entirely predictable, enhancement might exist secondary to contrast extravasation along the fresh surgical wound.

**Figure 3:**
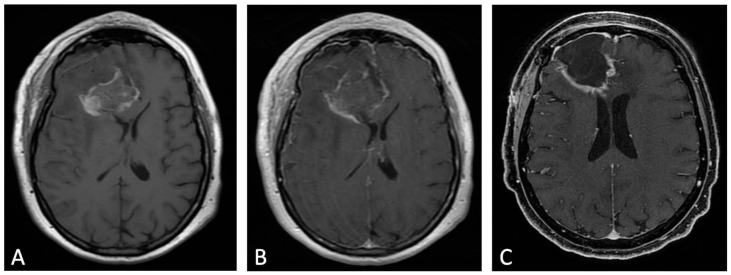
Example of evolution of thick linear CE. Pre-contrast (**A**) and post-contrast (**B**) T1w images of early post-operative MRI after surgical resection of GB; post-contrast T1w of MRI performed one month after surgery (**C**). Thick peripheral enhancement may be seen particularly along the medial and posterior margin of the surgical site, with obvious tumor progression consistent with an area of thick linear-nodular enhancements at the resection bed one month after surgery.

Contrast enhancement (CE) in early post-operative MRI can be classified in three different patterns, according to Ekinci et al. [9]: thin linear (like normal dural enhancement), thick linear or thick linear-nodular enhancement (thicker than typical dural enhancement or >5 mm, with or without nodularity, respectively), or mass-like (enhancement thicker than at least 1 cm in any imaging plane). The thin linear pattern represents an expected MRI finding, whereas thick linear-nodular enhancement and mass-like appearance should be carefully reported because the first is associated with tumor regrowth, and the latter represents residual tumor (Figure 3) [12]. Accordingly, Garcia-Ruiz et al. demonstrated that pathological enhancement thickness on post-surgical MRI correlated with both progression-free survival (PFS) and overall survival (OS) [16].

**Figure 4:**
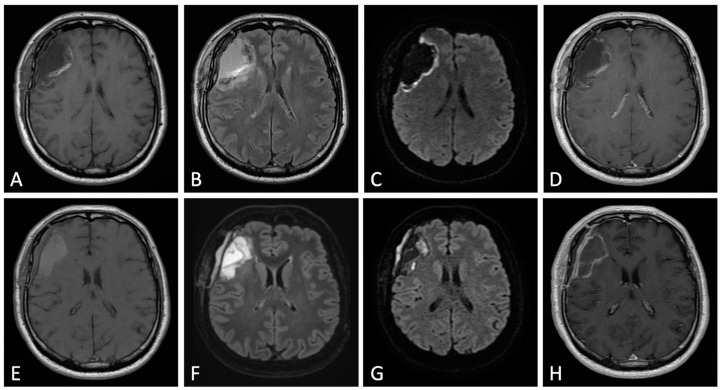
Differences between early (48 h, upper row) and delayed (one week, lower row) post-operative MRI. Axial pre-contrast T1w (**A**,**E**), FLAIR (**B**,**F**), DWI (**C**,**G**), and post-contrast T1w (**D**,**H**). One week after surgery, pre-contrast T1w image (**E**) shows marked T1 shortening caused by the formation of methemoglobin within the surgical bed. “Benign” widespread enhancement also occurs along the resection margins (**H**); it does not allow for the distinction of residual tumor from surgically induced enhancement.

MRI scans performed on post-operative days 4 to 21 are usually too late, suffering from marginal ‘reactive’ enhancement due to hypervascularization and disruption of the blood–brain barrier (BBB) during scar formation, as well as the presence of methemoglobin in the operative site (Figure 4) [17]. 

Moreover, during this period, about 10% of the patients show new and often gyriform enhancement of the adjacent parenchyma due to subacute ischemia. Post-operative benign enhancement could be observed for up to 3 months but almost never beyond. Only in few exceptional cases, it could persist up to 6 months after surgery. 

The role of advanced imaging modalities in the early post-operative MRI is strongly limited. DWI is undoubtedly the most used sequence and its role has been investigated. A thin rim of DWI hyperintensity around the tumor cavity is frequently seen and attributed to post-operative changes, while more extensive DWI bright signal beyond the tumor cavity margin (absent on the pre-operative scan) is considered new post-operative DWI lesion correlating with poor functional outcome [18,19].

DWI can prevent misinterpretation of CE on subsequent scans; regions interpreted as post-operative ischemia frequently show CE within 15–75 days post-surgery. A new enhancement observed after glioma surgery should be interpreted in the context of DWI obtained immediately post-operatively, allowing for the differentiation between CE related to post-operative infarct and tumor progression [20].

The use of PWI and MRS has been evaluated in clinical trials but remains technically challenging, and it is not currently part of standard practice. However, MRI performed within 48 h appears to improve the diagnostic accuracy of rCBV derived from DSC-PWI in identifying post-surgical residual GB [21]. Moreover, Lee et al. explored the use of DSC-PWI and DWI on early post-operative MRI study, demonstrating that normalized cerebral blood volume (nCBV) of enhancing areas on early post-operative MR imaging may be feasible for predicting GB response to CCRT with TMZ [22].

In a recent study Cui et al. explored, for the first time, the relationship between metabolic changes and tumor recurrence in the peritumoral zone in post-operative MR (within 48–72 h) in patients with GB [23]; they concluded that ratio of Cho/NAA ≥ 1.31 in the post-operative peritumoral zone predicts earlier recurrence and is associated with poor prognosis, allowing for more precise predictions of survival time and timely adjustments to therapeutic regimens.

Currently, to the best of our knowledge, ASL, PWI-DCE and DTI techniques in the early post-operative MRI have not been investigated.

## 3. MRI Findings during First-Line Therapy

### 3.1. Stupp Protocol

The current standard of care for GB is surgical resection followed by RT and concomitant and adjuvant TMZ chemotherapy, which is named Stupp protocol after the oncologist who proposed this treatment. Specifically, the protocol includes the administration of radiotherapy to the post-operative bed (total 60 Gy–2 Gy per daily fraction from Monday to Friday over 6 weeks), and TMZ during RT (75 mg/m^2^ of body surface area per day, 7 days per week) and post-RT after a 4 week-break (adjuvant, 6 cycles consisting of 150 to 200 mg/m^2^ of body surface area for 5 days during each 28-day cycle) [24]. 

TMZ is a second-generation alkylating chemotherapy agent absorbed after oral administration that crosses the BBB and which has a schedule-dependent antitumor activity. Its effect is based on the inactivation of MGMT (a DNA-repairing protein) and its subsequent activation of the p53 pathway that leads to apoptosis. Consequently, the identification of MGMT expression and p53 status in GB might help to identify those patients who will or will not respond to TMZ [25].

Although in individuals 70 years of age or younger, a standard Stupp protocol is usual, in older individuals the optimum treatment regimen is less well established, particularly in those with significant comorbidities. 

Despite this, even in the best-case scenario, GB has a poor prognosis with a median survival of <2 years [26].

### 3.2. MRI during Stupp Protocol

MRI is the modality of choice for the routine follow-up of GB during the Stupp protocol to monitor the possible appearance of pathological tissue in the surgical bed and other brain areas. As is known, the appearance of signal alterations on MRI during the treatment phase is not necessarily tumor tissue; it may be post-treatment changes or, more often, the coexistence of both [27,28].

During first-line therapy and its follow-up, treatment-related complications are mainly due to radiation injury, and it is possible to separate them on the basis of their time of occurrence in [29]:

- Acute and early delayed: days to months (usually less than 3 months) following treatment, generally transient (e.g., pseudoprogression);
- Late delayed: at least 6 months after radiation and considered irreversible and progressive (e.g., radiation necrosis).

Since both true progression (TP) and treatment-related alterations have BBB disruption and vasogenic edema, the resulting MR features are T2w/FLAIR (fluid-attenuated inversion recovery) hyperintensity and contrast-enhancing areas. Thus, it remains a challenge for the radiologist to distinguish between them, or, when both are present, which one gives the predominant component.

Macdonald criteria and RANO criteria (Table 2) [30,31], which are commonly used in assessing brain tumor progression, primarily rely on the size of the enhancing component on MRI scans. These criteria have limitations in accurately identifying true disease progression and differentiating it from post-treatment changes. In order to overcome these limitations, it is recommended to include advanced MRI sequences in the follow-up protocol for brain tumor patients. These advanced sequences, such as diffusion-weighted imaging (DWI), perfusion-weighted imaging (PWI), and magnetic resonance spectroscopy (MRS), provide additional information that can help non-invasively assess various aspects of the tumor and treatment effects [32].

DWI measures the diffusion of water molecules in tissues and can be useful in detecting areas of restricted diffusion, which may indicate active tumor or residual disease, as they suggest regions of higher cellularity [33]. PWI evaluates blood flow within the tumor, which can provide insights into tumor vascularity and help differentiate between treatment-related changes and tumor progression. MRS analyzes the chemical composition of tissues and can help identify the presence of specific metabolites associated with tumor cells.

By incorporating these advanced MRI sequences into the follow-up protocol, clinicians can obtain more comprehensive information about the tumor and its response to treatment. This can aid in distinguishing between post-surgical residual tumor, non-enhancing tumor, and treatment-related alterations, thereby improving the accuracy of disease assessment and progression monitoring.

### 3.3. Early Post-Treatment Alterations

#### 3.3.1. Pseudoprogression: Definition and Physiopathology

Pseudoprogression (PsP) is an early-delayed treatment-related alteration (first 3 to 6 months after completion of chemoradiotherapy), radiologically defined as new or enlarging contrast-enhancing areas on follow-up MRI, which subsides or stabilizes without further treatments [34].

It occurs in approximately 20–30% of patients, and it is thought to be due to inflammatory tissue reactions and oligodendroglial injury secondary to irradiation. It may be increased by TMZ, resulting in transient vessel dilatation and permeability, and vasogenic edema [11,34,35].

PsP may or may not be associated with neurological symptoms, and it is more common in patients with methylated MGMT-promoter GBs. Moreover, some studies suggest that PsP may have a relatively good prognosis [34].

#### 3.3.2. Imaging: Conventional and Advanced MRI Sequences:

As previously told, the hallmark of PsP is the presence of new or enlarging contrast-enhancing areas. By conventional MRI alone, it can be challenging to distinguish between TP and PsP. Advanced MRI techniques provide additional biomarkers that can improve diagnostic specificity. 

DWI definitely represents the most widely used and available technique, so that it is no longer considered as advanced. Through the evaluation of DWI signal and ADC maps, it is possible to obtain crucial information on the nature of parenchymal alterations. In particular, GB usually shows restricted diffusivity and decreased ADC values due to increased cellularity. On the contrary, PsP has elevated ADC values, mainly reflecting vasogenic edema. A mean ADC value lower than 1200 × 10^−6^ mm^2^/s is reported to be more suggestive of TP than PsP [34]. Moreover, due to the damage of white matter fibers, DTI (a technique based on the directional variation of water diffusivity) shows a reduction in FA values in PsP [34,36]. 

PWI often represents the key to the interpretation of MRI findings during follow-up of treated GBs. PWI includes different techniques, such as DSC and DCE (which exploit properties of exogenous gadolinium contrast medium), and arterial spin labeling (ASL), which is based on the magnetic labeling of inflowing arterial blood used as an endogenous contrast agent. Each technique has its strengths and limitations, and the choice of technique depends on factors such as the clinical question, availability of resources, and patient-specific considerations. 

**Figure 5:**
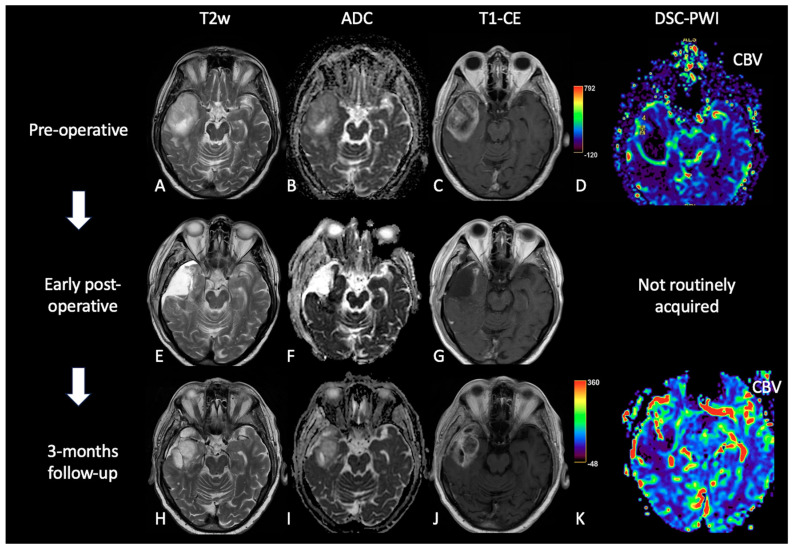
Case of PsP. (**A**,**E**,**H**) T2w axial images; (**B**,**F**,**I**) ADC maps; (**C**,**G**,**J**) post-contrast T1w; (**D**,**K**) DSC-CBV maps. The upper row shows a right temporal GB with low ADC (in its solid component), CE, and high perfusion values in the CBV map. In the middle row, there are post-operative images of macroscopically complete tumor resection. The lower row shows images two months after the beginning of Stupp treatment, highlighting the appearance of tissue with CE in the surgical bed, which shows increased ADC values compared to the primary tumor and without a significant increase of rCBV values, suggesting PsP.

**Figure 6:**
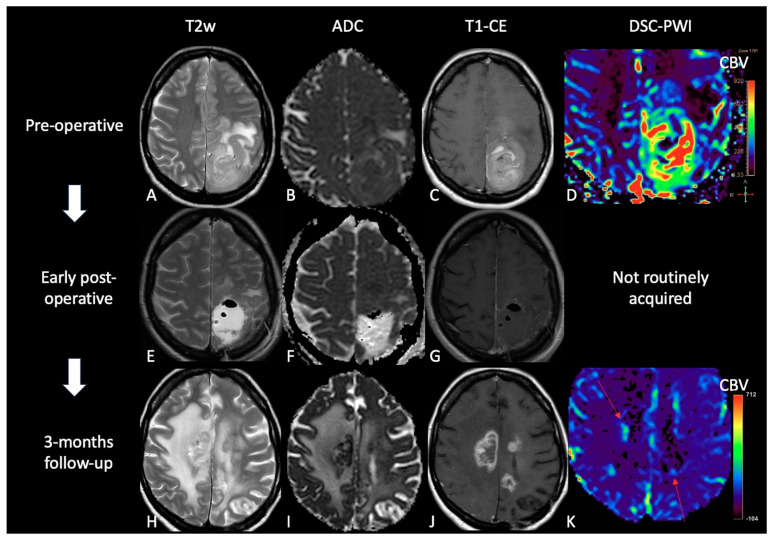
Case of TP. (**A**,**E**,**H**): T2w axial images; (**B**,**F**,**I**) ADC maps; (**C**,**G**,**J**) post-contrast T1w images after contrast; (**D**,**K**) DSC-CBV maps. The upper row shows a left parietal GB, with low ADC, CE, and high perfusion values in the CBV map. In the middle row there are post-operative images of macroscopically complete tumor resection. The lower row shows images three months after Stupp treatment, highlighting the appearance of tissue with CE in different site from the surgical bed, with low ADC values and high rCBV values, suggesting TP.

DSC is actually the most used and validated PWI technique. It is a T2*-weighted sequence that detects the susceptibility effects of contrast medium. Its main parameters are relative cerebral blood volume (rCBV), relative cerebral blood flow (rCBF), and mean transit time (MTT). Among these, rCBV (mean and maximum) allows for the discrimination of the areas of PsP (lower mean and maximum rCBV) from TP (higher mean and maximum rCBV) (Figure 5 and Figure 6) [34,37]. Unfortunately, there is no universally accepted cut-off value for rCBV to make this distinction, probably because the interpretation of rCBV values depends on various factors, including the imaging technique, sequence used, tumor type, and brain region involved. 

DCE is a T1-weighted technique that offers quantitative pharmacokinetic parameters of the tumor microcirculation structure, particularly permeability and perfusion. The main quantitative parameters include Ktrans (the volume transfer coefficient from the plasma to extracellular space), Ve (the fractional volume of extracellular-extravascular space), and VP (the fractional volume of total plasma), whereas semi-quantitative parameters are the initial area under the signal intensity-time curve (IAUC), initial slope of the curve, maximum enhancement value, and time to peak. DCE studies are less numerous than DSC, but have shown that patients with PsP had significantly lower Ktrans, Vp, and Ve values than patients with TP [37,38,39]. Regarding ASL, cerebral blood flow (CBF) is its most important parameter. Studies focusing on PsP specifically using ASL are scarce; in one study ASL shows similar specificity and sensibility to DSC in differentiating PsP (lower CBF) to TP (higher CBF) [40]. 

At MRS, TP is typically characterized by choline (Cho) elevation (marker of cell membrane turnover/cellular proliferation), N-acetylaspartate (NAA) reduction (marker of marker of neuronal integrity and viability), and high Cho/Cr and Cho/NAA ratios. Differently, PsP lacks a significant increase in Cho but usually shows a variable decrease in NAA and the presence of Lip peak (a marker of necrosis) [32]. Lower Cho/Cr and Cho/NAA ratios and an elevated lipid peak are suggestive of PsP. [26,34,41]. Moreover, a recent study describes the use of less common metabolites, such as myo-inositol (mI) and glutamate plus glutamine (Glx); particularly, TP shows higher Lac/Glx and lower mI/c-Cr (contralateral creatine) than PsP [26]. However, in some cases of TP, enhanced lactate and lipid concentrations may suppress the peaks of other metabolites, including Cho [42,43].

### 3.4. Late Post-Treatment Alterations

#### 3.4.1. Radiation Necrosis: Definition and Physiopathology 

Radiation necrosis (RN) is a late-delayed complication that typically occurs 6–24 months post radiotherapy but can occur up to several years/decades [34]. There is neither clear evidence of nor consensus on the distinction between PsP and RN, as the physiopathology of the radio-induced lesions is dynamic and complex [44].

RN affects 5–40% of patients, and it appears like a space-occupying mass with associated neurological symptoms. Its physiopathological mechanisms are not clear but probably rely on vascular endothelial damage, glial and white matter damage, and the activation of the fibrinolytic enzyme system, which lead to cytotoxic and vasogenic edema, demyelination and tissue necrosis [28,34,44]. Main histopathological findings are vascular dilation and telangiectasias, wall-thickening and vessel hyalinization, fibrinoid necrosis of blood vessel walls, and adjacent perivascular parenchymal coagulative necrosis [28,34].

#### 3.4.2. Imaging: Conventional and Advanced MRI Sequences

The distinction between RN and TP is often not possible with conventional MRI alone. More typical morphological features of RN are the “Swiss cheese” CE pattern and the involvement of septum pellucidum, whereas progressive enhancing enlargement with mass effect and involvement of the corpus callosum are more suggestive of TP. However, RN and TP can mimic each other and often coexist. Therefore, advanced MRI techniques are required to correctly interpret MRI findings [45,46].

DWI and DTI have been assessed to differentiate TP and/or residual tumor from RN; ADC values were noted to be higher in RN than in TP and like in PsP, some studies have demonstrated lower FA values in RN than in TP [34,47].

**Figure 7:**
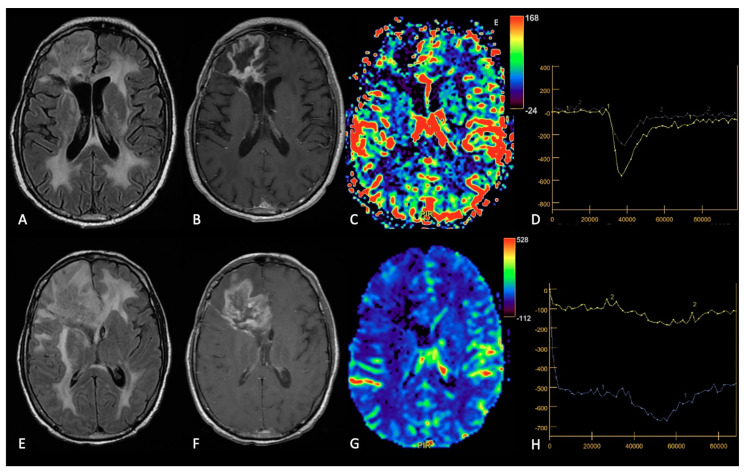
Example of RN. FLAIR (**A**,**E**), post-contrast T1w (**B**,**F**), DSC-CBV maps (**C**,**G**) and DSC- signal intensity/time curves (**D**,**H**).The upper row shows a case recurrent GB, with enhancing tissue surrounding the surgical cavity and increased rCBV values, without significative mass effect.After surgery and radio-chemotherapy, follow-up MRI (lower row) shows a large enhancing lesion with extensive vasogenic edema and mass effect, but without increased rCBV values. After another surgery, the histological showed radiation necrosis without tumor recurrence.

About PWI, rCBV value is actually the most used advanced MRI indicator in post-treatment tumor assessment; indeed, several studies have shown that rCBV is lower in RN than in TP (Figure 7) [34]. Similarly, Ktrans is significantly lower in RN than in TP too [48].

Patel et al. performed a systematic review and meta-analysis to evaluate DSC and DCE in differentiating recurrent glioma from post-treatment changes, including both PsP and RN. The results showed that for DSC, the pooled sensitivity was 90% and the specificity was 88%. For DCE, the pooled sensitivity was 89% and the specificity was 85% [49]. ASL is rarely used in this context, but one study confirmed that the normalized ASL-CBF ratio was significantly higher in TP than in RN [50]; there are also other studies that depict its usefulness in distinguishing RN from TP [47,51].

MRS results are less specific, as there could be an overlap between metabolic alterations in TP and RN. The best parameters to distinguish between these two entities are Cho/Cr and Cho/NAA ratios, which are significantly higher in TP than in RN [26,34,41,52].

**Figure 8:**
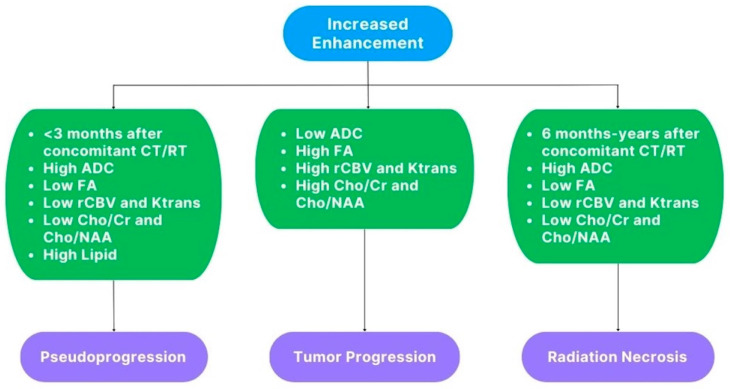
Schematic representation of the main “advanced” findings in PsP, TP, and RN.

Below, we propose a summary scheme of the main “advanced” features of pseudophenomena and TP for a practical differential diagnosis between them (Figure 8).

## 4. MRI Findings during Second-Line Therapy 

Therapeutic choices in recurrent GB are still debated, and no data-driven guidelines are available to ease clinical decisions. Some treatment options have been proposed, including re-operation, re-irradiation, and systemic therapy, alone or in combination [53,54], but the choice of second-line treatment depends on several factors, such as the patient’s overall health, the time to disease recurrence, the location, size and extent of the recurrent tumor, the response to previous treatments, the molecular and methylation status of the tumor, and the toxicity profile of the drug [54].

Usually, in the case of local recurrence, surgical resection precedes chemotherapy and less frequently re-irradiation, while in case of diffuse recurrence, systemic therapy is the first choice [7,54,55].

The available systemic treatments range from the oldest and commonly used chemotherapeutic agents, including lomustine (CCNU) given as a single agent or given in combination (PCV regimen) or re-challenge with TMZ [56,57], to novel targeted antiangiogenic and anti-growth-factor agents (e.g., bevacizumab and regorafenib) [58,59,60,61]. However, to date, their effectiveness in terms of length of OS remains still debated. 

The effects of treatment impact on radiologic phenotypes and the interpretation of MRI abnormalities following the treatment failure remains the main diagnostic challenge. In addition, the introduction of novel antiangiogenic and anti-growth factor agents, such as bevacizumab (BEV) and regorafenib (REG), showed peculiar MRI patterns that required a revision of RANO criteria, including T2w/FLAIR lesions as a new criterion for glioma progression [62]. However, the revised evaluation criteria, based on conventional MR imaging alone, has also shown its weaknesses in differentiating non-enhancing progressive tumors from other causes of hyperintensity in T2w/FLAIR sequences, such as vasogenic edema, leukoencephalopathies, and microvascular ischemic spots. Advanced MRI sequences (DWI, PWI, and MRS) have demonstrated their usefulness in overcoming this problem, improving the assessment of treatment response in GB, by extending the existing RANO criteria [63]. 

The drug-related MR features/patterns of recurrence are grouped in two main categories according to currently approved treatments: traditional chemotherapeutic agents (e.g., TMZ, lomustine, and PCV) and novel targeted antiangiogenic agents (BEV and REG). 

In the former group the recurrence pattern looks like the common MRI features of disease progression: increased T2w/FLAIR signal abnormality (due to edema and tumor infiltration) and increased CE according to standard criteria RANO, restricted DWI, and increased rCBV. On the other hand, the novel therapies, at the first follow-up, led to a dramatic reduction in the tumor CE as well as reduction in edema on MRI (“called” pseudoresponse) and, at progression, to an increase in non-enhancing T2w/FLAIR abnormalities. In this latter group, these effects are due to the stabilization of the immature and friable vasculature of the tumor and the decrease in the rate of microvascular proliferation and the effects on BBB permeability [64].

However, although the patterns of recurrence of novel targeted antiangiogenic agents (BEV and REG) may look similar, they differ because of their different ways of action. 

BEV is a humanized monoclonal antibody directed against vascular endothelial growth factor (VEGF) [65,66], while REG is an orally available multikinase inhibitor with several molecular targets involved in angiogenesis (VEGFR1–3 and TIE2), oncogenesis (KIT, RET, RAF1, and BRAF) and maintenance of the tumoral microenvironment (PDGFR and FGFR) [67,68,69]. Their distinctive patterns may be appraisable by using combined standard and advanced MRI modalities, and below, the main specific MR patterns of failure under BEV and REG treatment are reported. 

### 4.1. Bevacizumab

At present there is no general agreement on the definition and categorization of recurrence patterns following treatment with BEV, and it is probably due to the biological features and the different resistance mechanisms of tumor to BEV. Patterns of disease recurrence have been recently characterized by Pope from the prospective BRAIN trial and by Nowosielski in single institution retrospective reviews [64,70,71].

**Figure 9:**
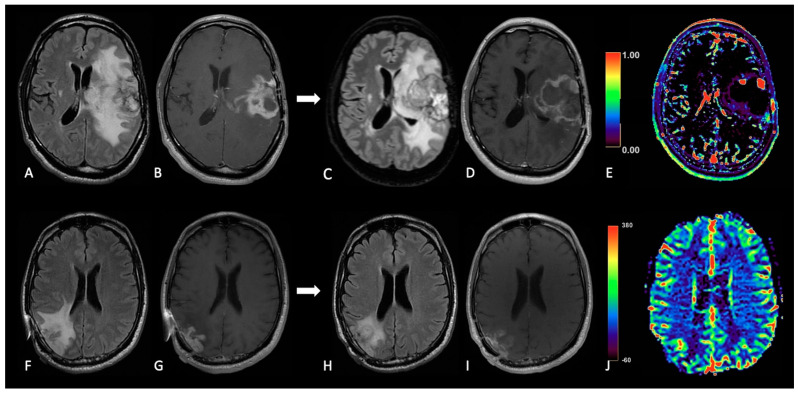
TP and pseudoresponse after BEV. Upper row: MRI at baseline and after 6 months of BEV. FLAIR (**A**,**C**), post-contrast T1w images (**B**,**D**), and DCE-Ktrans map (**E**). At follow-up there are increase in FLAIR signal intensity and enhancing tumor components, with high values of Ktrans in the solid components, suggestive of TP. Lower row: MRI at baseline and after 5 months of BEV. FLAIR (**F**,**H**), post-contrast T1w images (**G**,**I**) and DSC-CBV perfusion map (**J**). Follow-up MRI shows the reduction in FLAIR signal intensity and CE, with high rCBV values, characteristics of pseudoresponse.

To define the categories of relapse Pope et al. focused on the location and enhancement of recurrent disease and described patterns of local, diffuse, distant and multifocal recurrences from the original tumor site [68]. Nowosielski et al. analyzed CE development and T2 hyperintense signal changes during treatment and classified five groups of patterns: two types of a solely T2-based tumor progression (“T2 diffuse” and “T2 circumscribed”), two types of T1 contrast enhancing phenotypes (“cT1 relapse” characterized by a complete disappearance of CE during therapy and relapse at progression and “classic T1” characterized by an incomplete decrease in CE during treatment and CE increase at progression), and a “primary non-responder” with no decrease in CE or development of new lesions at first follow-up imaging [64]. It is well described that BEV treatment is associated with restricted diffusion detected at 1–6 months from treatment, but the significance of these lesions is controversial because DWI lesions may reflect both increased tumor cellularity, then predictive of recurrent tumor, and BEV-induced cytotoxic edema (cell death/necrosis), then without tumor progression [72]. On the other hand, at follow-up after antiangiogenic treatment, a high nCBV may be predictive of future local enhancing progression (Figure 9) [73].

### 4.2. Regorafenib

Very few studies described radiographic patterns following REG therapy, because only a few REG cases were reported in the literature. Available studies described only two MRI progression patterns, named patterns A and B [74,75]. 

Pattern A is similar to classic progression disease reported by increasing CE and increasing T2w/FLAIR signal abnormality; moreover, stable or new sporadic hyperintensity dots on DWI/ADC (due to tumor cellularity, ischemia, cell death, necrosis or hemorrhage), increasing number of intratumoral black dots (due to hemorrhage), and stable or high rCBV (due to neovascularization) compared to previous MRI have been reported.

**Figure 10:**
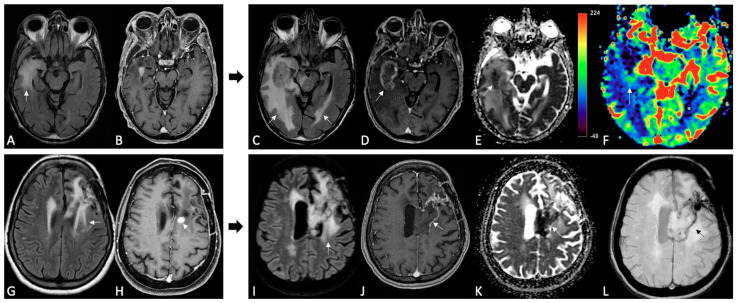
MRI changes in recurrent GB under regorafenib, patterns A and B. MRI scans were performed at baseline (**A**,**B**,**G**,**H**) and 3 months after the first administration of REG therapy (**C**–**F**,**I**–**L**). FLAIR (**A**,**C**,**G**,**I**), post-contrast T1w (**B**,**H**,**D**,**J**), ADC map (**E**,**K**), DSC-CBV map (**F**) and SWI (**L**). Pattern A (upper row). The 3-month follow-up showed an increase in size, more than 50%, of FLAIR signal intensity and enhancing tumor in the right temporal lobe. Note the focal diffusion restriction on the ADC map and high rCBV within the enhancing areas. Pattern B (lower row). On 3-month follow-up MRI, the previously enhanced tumor component showed a dramatic reduction in the CE area with evidence of only subtle peripheral enhancement. Also, note the slight increase in FLAIR signal intensity, diffusion restriction on the ADC map (involving the corpus callosum) and a thin hypointense rim on SWI.

Pattern B includes most features of the “T2-dominant growth pattern” coined for BEV, characterized by decreasing CE and increasing (relative or absolute) T2w/FLAIR hyperintensity. The main findings of pattern B were the marked hyperintensity on DWI of tumor components showing decreased signal intensity on T2w and marked decrease in CE of the target lesions with marginal or dot-like enhancing component, residual or of new-onset. Reduced rCBV in the DWI hyperintensity components and hypointense rim on susceptibility-weighted imaging (SWI) surrounding the hyperintense tissue on DWI were also reported (Figure 10).

There is no accordance about interpretation of MRI modifications caused by REG: diffusion restriction has been explained by coagulative necrosis or constant hemorrhagic diapedesis [76], whereas the surrounding hypointense thin rim on SWI as a combination of causes including granulation tissue, blood product, or neuroinflammatory process free radicals [77,78]; moreover, the reduction in CE and the hypoperfusion detected at PWI have been associated with antiangiogenic action of the drug that prevents the development of a rich vascular system with regression of hypervascularization in the tumor [79].

## 5. Radiomics and Artificial Intelligence in Treated GB

### 5.1. Introduction

Radiomics is a promising approach that can contribute to precision medicine by quantitatively analyzing clinical imaging arrays and utilizing artificial intelligence (AI) methods to improve the objectivity, accuracy, and the automation of radiological diagnoses. Machine learning (ML), a subfield of AI, can create computational models that can achieve astonishing results in aiding clinical decisions, by training the model with datasets.

Due to limited access to private and customized high-quality labeled brain tumor datasets, which are typically owned and protected by medical institutions, public datasets play a crucial role in providing an equal platform for ML researchers to train and compare the outcomes of their models. In the field of neuro-oncology, one of the most widely used public image datasets is the Brain Tumor Segmentation (BraTS) challenge, organized by the Medical Image Computing and Computer Assisted Interventions (MICCAI) and other professional organizations since 2012. As of July 2023, the latest version, BraTS 2023, comprises more than 4500 brain tumor cases/patients, divided into three subsets: training, validation, and testing. Only the training and validation subsets are publicly accessible, and they include multimodal 3D MRI scans (T1, T1-CE, T2w, FLAIR) for each case. Other commonly used datasets include The Cancer Imaging Archive (TCIA) and The Whole Brain Atlas by Harvard Medical School [80].

Most AI techniques employed in brain tumor radiomic studies utilize supervised ML, which trains a model to predict a target variable from a set of predictive variables (data samples, taken from private or public databases), with the help of labels/annotations and a loss function (a mathematical function that measures the error between predicted and actual values in a machine learning model, and so how well the algorithm works; during training, the aim is to minimize this “loss” between the predicted and target outputs).

Various ML models have been employed for brain tumor radiomic analysis, such as support vector machines (SVM), k-nearest neighbors (k-NN) and decision trees (DTs).

**Figure 11:**
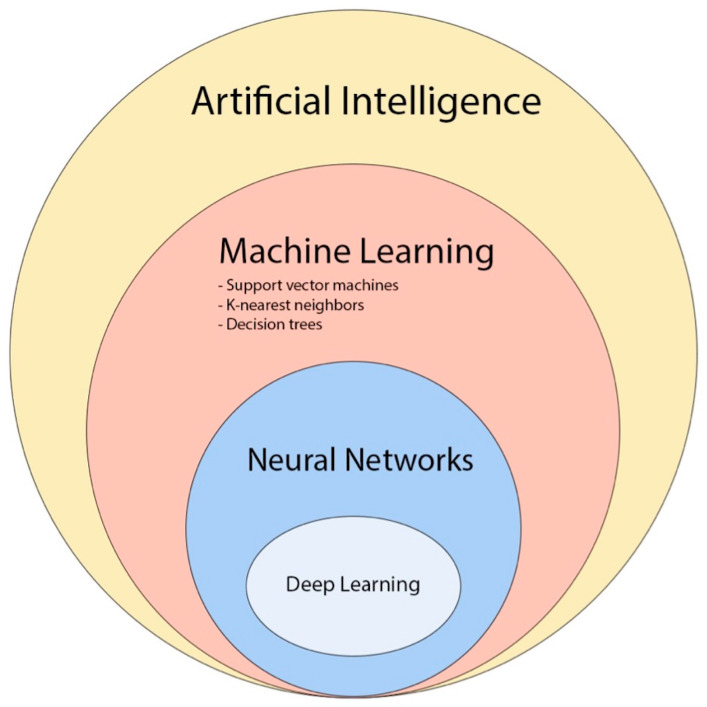
The diagram provides an overview of the various concepts and subfields related to artificial intelligence and how they are related with each other [81].

Recently, convolutional neural network (CNN)-based deep learning (DL) has gained popularity in neuro-oncology imaging due to its scalability and ability to extract local and global features (Figure 11) [80].

A possible issue of this approach is that current mathematical mechanisms utilized in ML models rely on statistics, which implies that there might not exist a deterministic optimal algorithm to achieve optimal outcomes. Even when employing public datasets like BraTS, the initial values of trainable parameters in ML models, as well as subtle structural differences, can have a significant impact on the training results and can yield diverse outcomes. Consequently, some researchers resort to simply adding more layers to CNNs in an attempt to enhance accuracy. However, this approach can introduce excessive and unnecessary computational complexity during the training process, disregarding the biological connections and underlying meaning of the data and making the classifier potentially inefficient. Conversely, excessive engineering efforts in data pre-processing, such as strong feature extraction and data restriction or collection, can lead to the overfitting of the ML network to the training data, resulting in the loss of generalizability for larger populations with greater diversity, therefore limiting its clinical relevance [80].

**Figure 12:**
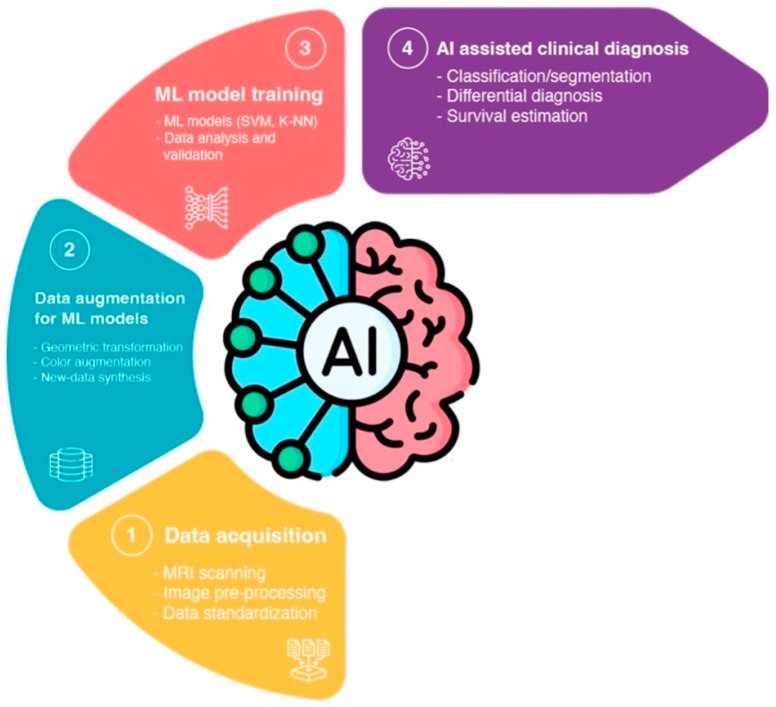
Workflow of AI-assisted GB analysis: (1) Acquisition of radiological image data through MRI scanning of GB patients. The images undergo pre-processing and standardization before being labeled by radiologists. (2) Data augmentation and pre-processing for ML models involves the use of different techniques to enhance data generalizability. The data undergo optional pre-processing for ML modeling, including feature extraction to extract explicit features and filter out unnecessary data. (3) ML modeling and training involve feeding the augmented and pre-processed data into different ML models for GB radiomic analysis training and validation. (4) AI-assisted clinical diagnosis/deployment involves predictions from the ML models for different medical goals, such as differential diagnosis and survival estimation [80].

The process of a ML-based GB radiomic analysis can be broken down into four major steps. Firstly, data acquisition involves performing MRIs on patients with a brain tumor, pre-processing the raw MRI data, and having radiologists labeling the data by radiologists to define the regions for the ML training and validation process. Secondly, data augmentation for ML models involves processing image data in pair-wise format to increase sample variety, using approaches such as geometric transformation, color augmentation, and synthesis of similar-appearing imaging data. The augmented imaging data is then pre-processed through feature extraction to simplify and improve the effectiveness and efficiency of the subsequent ML training process. Thirdly, the training and validation of the ML model, which involves feeding the augmented and pre-processed data to ML models in order to train them much more effectively. Finally, the trained models can be deployed to perform predictions, such as classification and segmentation for AI-assisted clinical diagnosis/deployment (Figure 12) [80].

### 5.2. TP vs. Treatment-Related Changes

In the last decade, ML algorithms achieved interesting results that may aid in the follow-up management of treated GB patients in the near future and, more specifically, in differentiating treatment effects versus recurrent disease/true progression and in OS prediction.

As said, the interpretation of conventional MRI may pose a challenge in distinguishing tumor recurrence from RN and PsP, but an accurate differentiation between these entities is critical for treatment decisions during follow-up. Advanced MRI modalities are useful, but they are not always available, and their processing is time consuming and requires expertise. From this point of view, AI can be a tool to arrive at a faster and more objective diagnosis.

Booth et al. used topological descriptors called Minkowski functionals to analyze tumor heterogeneity in T2 MRI and then utilized an SVM model, along with image features, such size and signal intensity, to distinguish between PsP and TP [82]. They achieved an accuracy of 0.88, with an AUC of 0.9. Hu et al., used T1 MRI and eight-dimensional feature vectors, including T2, FLAIR, ADC, PWI, derived rCBV, and relative cerebral blood flow (rCBF), to train an SVM model and achieved an AUC of 0.94 in distinguishing between PsP and TP [83].

Ismail et al., employed an SVM classifier and extracted 30 global and local shape features from T1-CE, T2, and FLAIR images to achieve an accuracy of 0.90 in distinguishing PsP from TP [84].

Tiwari et al. developed an algorithm on SVM classifiers trained to recognize a total of 119 radiomics features on three sequences (T1-CE, T2, and FLAIR) [85]. The algorithm performance was compared with that of two neuroradiologists, who evaluated the same patients using the three aforementioned sequences. The algorithm had superior diagnostic accuracy to the experts in distinguishing RN form TP, in particular the radiomics data collected on the FLAIR sequences proved to be the most significant, with an AUC equal to 0.79.

Park et al., developed a ML algorithm using SVM, k-NN, and AdaBoost and took into account 263 radiomics parameters obtained on T1-CE, T2, and ADC maps [86]. The model trained on ADC maps obtained the best diagnostic results in the differentiation of RN from TP, with an AUC, accuracy, sensitivity, and specificity of 0.80, 78%, 66.7%, and 87%, respectively.

### 5.3. Overall Survival

Radiomic analysis has been shown to also provide more objective and accurate prediction of disease prognosis compared to conventional survival prediction based on clinical information, which is of great clinical importance and could benefit both treatment planning and patient care. Sanghani et al. utilized an SVM ML model to analyze texture, shape, volumetric features, and patient age derived from multimodal MRI data (T1-CE, T2, and FLAIR) of 173 patients to perform binary and multiclass OS prediction with accuracies of 0.987 and 0.89, respectively [87]. 

In another study, Nie et al. proposed a 3D deep learning model using a CNN to extract features from multiparametric maps computed from multimodal multichannel MRI (T1-CE, DTI, and resting state-fMRI), demographic, and tumor-related features to train an SVM to predict the OS (long or short overall survival time), achieving an accuracy of 0.91 [88]. These findings demonstrate the potential of radiomic analysis to provide beneficial information for personalized treatment.

### 5.4. Prediction of Tumor Invasion and Recurrence

Another growing subfield of AI application in GB and high-grade gliomas (HGG) is the prediction of tumor invasion and recurrence.

One of the difficulties in treating GB lies in the inability to detect the cancer’s invasive region beyond the contrast-enhancing tumor, since neoplastic cells infiltrate the non-enhancing peritumoral area, leading to high rate of local progression. Recent evidence suggests that extending the surgical resection of GB beyond the contrast-enhancing region could enhance patient survival. However, enlarging surgical margins may not always be feasible due to the risk of post-operative neurological deficits when the peritumor extends to critical areas [89]. The challenge arises from the fact that conventional MRI often fails to visually differentiate a non-enhancing tumor from vasogenic edema, despite specific radiological criteria. AI could help radiologists in this distinction, thanks to its ability to see changes non perceptible to the human eye, and thus can lead to new and targeted surgical limits and targets for radiotherapy.

To address this issue, Cepeda et al. conducted a study utilizing a machine learning-based approach to predict tumor recurrence in GB patients. Their method involved analyzing radiomic features extracted from post-operative MRI scans from a cohort of 55 patients. The ground truth for tumor recurrence was determined based on follow-up MRI scans, while voxel-based radiomic features were extracted from the post-operative structural multiparametric MRI scans. They trained a total of four machine learning-based classifiers voxel by voxel, and the Categorical Boosting (CatBoost) classifier demonstrated the highest performance on the testing dataset, achieving an average area under the curve (AUC) of 0.81 ± 0.09 and an accuracy of 0.84 ± 0.06 using region-based evaluation [90].

The post-operative MRI radiomic feature extraction may be the key to this model’s promising results. Numerous studies have already developed ML models trained on radiomic features extracted from the pre-operative MRI, but this approach may have some intrinsic limits, such as predicting regions that have already been resected. Moreover, post-operative and follow-up MRI scans have more similarities in morphology and segmentation, resulting in more precise overlapping regions.

Yan et al., developed a recurrence prediction model utilizing voxel-based radiomic features from pre-operative MRI, including structural, perfusion, and DTI [91]. The authors reported an overall accuracy of 0.78 in the validation group.

Another example is the study by Rathore et al. [92], in which the authors included patients with confirmed recurrence diagnoses based on pathology and employed pre-operative MRI for defining ground truth labels, achieving an AUC of 0.91 and an accuracy of 0.89 in the test cohort. Even though these studies obtained remarkable results, they both possess the limitations discussed earlier.

## 6. Conclusions

The follow-up of treated GBs remains a challenge for neuroradiologists due to several factors. Firstly, there is a lack of standardization regarding the optimal timing of follow-up imaging.

Additionally, the effects of multiple therapies can overlap, making it difficult to differentiate between treatment-related changes and tumor progression or recurrence. This complexity necessitates a comprehensive evaluation of MR findings beyond morphological MRI alone. Advanced MR modalities may be required to provide a more accurate interpretation of post-treatment changes. However, these advanced techniques are not always readily available, and they can be susceptible to artifacts resulting from surgical interventions or previous treatments.

Furthermore, interpreting the results of advanced MR modalities requires expertise and experience. The nuances and potential pitfalls associated with these techniques make it necessary for neuroradiologists to possess deep knowledge and proficiency for proper interpretation. Artificial intelligence (AI) has the potential to assist in the analysis of MR data and aid in the identification of treatment-related effects. However, the integration of AI into clinical practice for GB follow-up requires careful validation and ongoing refinement.

In the era of precision medicine, a multidisciplinary team approach is crucial for the effective management of patients with treated GBs. Neurologists, neurosurgeons, radiation oncologists, and neuroradiologists need to collaborate closely to ensure the most appropriate treatment decisions for each individual case. This interdisciplinary approach allows for a comprehensive evaluation of clinical and radiological data, taking into account the specific treatment modalities employed and potential treatment-related effects.

In summary, the follow-up of treated GBs poses challenges to neuroradiologists due to the lack of standardization, the overlapping effects of multiple therapies, the limitations of morphological MRI alone, and the need for expertise in advanced MR modalities. The integration of AI and a multidisciplinary team evaluation can contribute to a more precise clinic-radiological assessment, aiding in the management of patients with treated GBs.

## Figures and Tables

**Table 1:** Summary of preferred and other possible regimens in GB [1].

Clinical Timepoint	Preferred Regimens	Other Recommended Regimens	Useful In Certain Circumstances
Adjuvant Treatment, KPS ≥ 60	RT + concurrent and adjuvant TMZ ± TTF	None	TMZ (for patients with MGMT promoter-methylated tumors and age > 70 years) RT + concurrent and adjuvant lomustine and TMZ (for patients with MGMT promoter-methylated tumors and age ≤ 70 years (category 2B)
Adjuvant Treatment, KPS < 60	None	None	RT + concurrent or adjuvant TMZ (for patients with age ≤ 70 years) TMZ (for patients with MGMT promoter-methylated tumors)
Recurrence Therapy	Bevacizumab TMZ Lomustine or carmustine PCV Regorafenib	Systemic therapy + bevacizumab ○ Carmustine or lomustine + bevacizumab ○ TMZ + bevacizumab	If failure or intolerance to the preferred or other recommended regimens ○ Etoposide (category 2B) ○ Platinum-based regimens (category 3) NTRK gene fusion tumors ○ Lerotrectinib ○ Entrectinib BRAF V600E activation mutation ○ Dabrafenib/trametinib ○ Vemurafenib/cobimetinib

**Table 2:** Sum up of Macdonald criteria and RANO criteria.

Response	Macdonald Criteria	RANO Criteria
Complete response	All: complete disappearance of all enhancing measurable and non-measurable diseases sustained for at least 4 weeks; no new lesions; no corticosteroids; being stable or improved clinically	All: T1-gadolinium enhancing disease: none; T2w/FLAIR: stable or decreasing; new lesion: none; corticosteroid: none; clinical status: stable or improving
Partial response	All: 50% or more decrease in all measurable enhancing lesions sustained for at least 4 weeks; no new lesions; stable or reduced corticosteroid dose; being stable or improved clinically	All: T1-gadolinium enhancing disease: ≥50% decrease; T2w/FLAIR: stable or decreasing; new lesions: none; corticosteroids: stable or decreasing; clinical status: stable or improving
Stable response	All: being not qualified for complete response, partial response or progression; being stable clinically	All: T1-gadolinium enhancing disease: >50% decrease but <25% increase; T2w/FLAIR: stable or decreasing; new lesions: none; corticosteroids: stable or decreasing; clinical status: stable or improving
Progression	Any: 25% or more increase in enhancing lesions; any new lesion; clinical deterioration	Any: T1-gadolinium enhancing disease: ≥25% increase; T2w/FLAIR; new lesions: yes, corticosteroids: not applicable

Abbreviations: RANO, Response Assessment in Neuro-Oncology; FLAIR, Fluid-attenuated inversion recovery.

## Data Availability

Not applicable.
